# Antifebrine or Acetanilide

**Published:** 1887-03

**Authors:** 


### *ANTIFEBRINE OR ACETANILIDE.*

Antifebrine, the latest antipyretic, is prepared by heating aniline with glacial acetic acid in a special receptacle. It is a white, neutral, crystalline body, without odor, with a slightly pungent taste, freely soluble in alcohol and hot water, but almost insoluble in cold water. It does not combine with acids or bases, and is non-poisonous, even in comparatively large doses. As an antipyretic it is four times more powerful than antipyrin.

In a series of observations made by Drs. A. Cahn and P. Hepp, assistants in Prof. Kussmaul's clinic, at Strasburg, it was found to possess remarkable power in controlling abnormally high temperatures, in cases of typhoid fever, erysipelas, acute rheumatism, pyæmia, pneumonia, etc. With the lowering of the temperature there was also a decrease in the frequency of the pulse, but an increase in volume It never caused any nausea, and the diaphoresis was always moderate in quantity.

These observers administered it in doses of four to fifteen grains, in brandy, wine, or wafers. They never exceeded a dose of thirty grains. The action of the medicine is manifest within an hour. They highly recommend it on account of its energetic action in small doses, also for its harmless effects on the stomach and for its cheapness. M. Lepine finds that it is a very valuable "nervine" in doses of seven and a-half grains, producing marked beneficial effects on the lightning pains in locomotor ataxia.

The Paris correspondent of the *Lancet* reports a recent discussion by the Societe de Therapeutique of antifebrine as a "nervine." Dujardin-Beaumetz claimed that it acted as a moderator of the spinal excitability, particularly of the upper part of the cord. No appreciable physiological effects were observed from daily administrations of fifteen to thirty grains. It cannot be detected in the urine. In three cases of locomotor ataxia the lightning pains were greatly relieved. In one case of epilepsy, the attacks had ceased entirely since its administration. M. Constantin Paul had tried antifebrine in doses of four to eight grains daily, as recommended by Lepine, but without result.—*Southwestern Medical Gazette.*

